# On the front line of modern data-management and Open Access publishing: Two years of PhytoKeys – the fastest growing journal in plant systematics

**DOI:** 10.3897/phytokeys.19.4501

**Published:** 2012-12-18

**Authors:** W. John Kress, Sandra Knapp, Pavel Stoev, Lyubomir Penev

**Affiliations:** 1 Smithsonian Institution, Washington DC, USA; 2 Department of Life Sciences, The Natural History Museum, Cromwell Road, London SW7 5BD, United Kingdom; 3 Bulgarian Academy of Sciences & Pensoft Publishers, Sofia, Bulgaria

“PhytoKeys has been among the first decided

*supporters of the open-access idea*,

and it has the experience, structures and wits

to combine speed of publication, high quality

standards and sophisticated editing techniques.”

Werner Greuter

OPTIMA *Newsletter* No. 40

PhytoKeys was launched on the 1^st^ of November 2010 as a novel, peer-reviewed, open-access outlet for plant biodiversity research (Penev et al. 2010a). The journal quickly gained the support of the international botanical community and since its launch continues to grow in reputation and volume.

The journal implemented several innovative technologies, such as a domain specific XML markup based on the TaxPub Schema (Catapano 2010; Penev et al. 2010b, Penev et al. 2011), compliant to the PubMedCentral standards, data publishing, automated export of content through web services to various aggregators, such as the Encyclopedia of Life (EOL), the Global Biodiversity Information Facilities (GBIF), Species-ID, bibliographic indices and so on (see e.g., Kress and Penev 2011). PhytoKeys became one of the very few journals in plant systematics to be accepted for coverage and archiving in PubMedCentral. The journal was the first to implement mandatory registration of new species and nomenclature changes with the International Plant Name Index (IPNI). PhytoKeysis CrossRef-compliant and plans also to use ORCID authors’ registry once it becomes fully operational, hopefully in 2013. The data publishing workflow in PhytoKeys is already integrated with the Dryad Data Repository and GBIF.

PhytoKeys was the first journal to announce the revolutionary changes instituted in the International Code of Nomenclature for algae, fungi and plants (ICN) in a paper published during the actual proceedings of the XVIII International Botanical Congress in Melbourne in July 2011, bringing the news to a world-wide audience (Miller et al. 2011). Thanks to the decisions of the Nomenclature Section of the Congress (see Knapp et al. 2011a), electronic publication of new taxa was allowed and PhytoKeys was again the first journal to demonstrate an immediate implementation of the Congress decisions, starting on the 1^st^ of January with a series of exemplar papers (e.g., Vorontsova and Knapp 2012, Tepe et al. 2012, Thomas et al. 2012), each paper with its individual publication date. The completed journal issue was printed on paper on the 7th of January 2012, demonstrating our commitment of the multiple archiving of botanical content.

**Figure 1.**
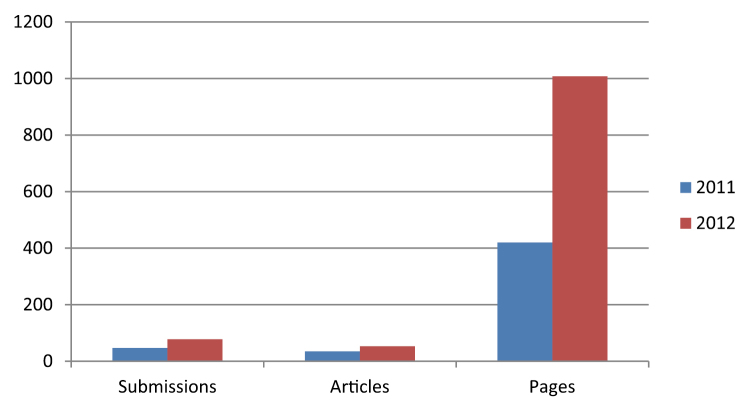
Total number of submitted manuscripts, published articles and pages per year in PhyltoKeys.

Since its launch the journal has received 130 submissions in total. Out of this number, 90 articles (1,456 pages) were published in volumes 1–18. Twenty-one manuscripts have been archived by the system because they were rejected during the review process, withdrawn from consideration or cancelled for other reasons. The yearly growth in numbers of published articles, issues, and number of pages in PhytoKeys has been substantial (Table 1; Fig. 1). The growth in number of published pages in the second year of PhytoKeys exceeds by 120% that of year 1. Starting with 35 articles in 2010–2011, in the second year the journal showed a substantial growth in submissions (66%) and published articles (50%). Based on the analysis of 30 randomly selected papers, the average publication time (from submission to publication) for the first two years of the existence of the journal is 78 days. The period between submission and acceptance is 63 days, and from acceptance to publication 15 days.

**Table 1.** Total number of submitted manuscripts, published articles, issues, and printed pages for the first two years of PhytoKeys.

**Year**	**Submissions**	**Published articles**	**Issues**	**Pages**
1 November 2010-31 October 2011	47	35	6	420
1 November 2011-31 October 2012	78	53	12	1,008
Total (until 5 December 2012)	130	90	18	1,456

**Figure 2.**
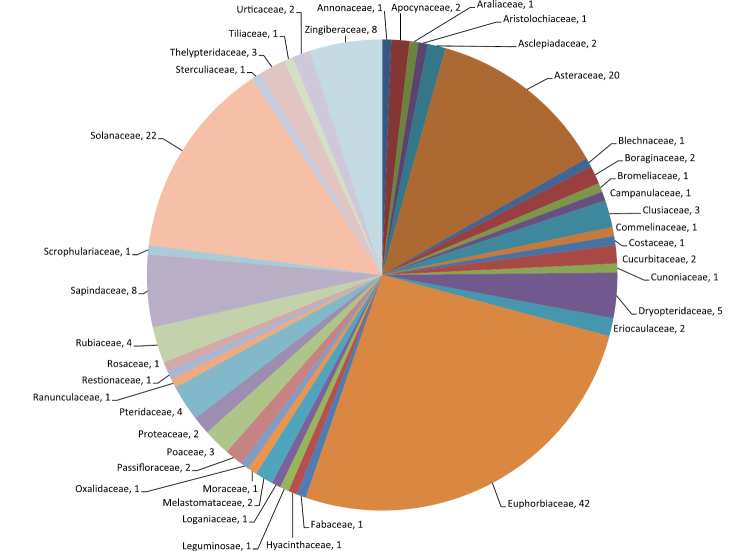
Taxonomic distribution by family of the published nomenclatural novelties in PhytoKeys.

Altogether, four new genera, one subgenus, 90 species, and one variety have been published in the journal since its launch, or 96 new taxa in total (Table 2). The number of new combinations (55) and new names (10) published for the same period is considerable (Table 3). Thus, the number of all nomenclatural novelties published in PhytoKeys for this short period totals 161. These new taxonomic contributions encompass 42 vascular plant families, with a predominance in the families Euphorbiacea, Solanaceae and Asteraceae, with 42, 22, and 20 contributions, respectively (Fig. 2).

**Table 2.** New taxa published in PhytoKeys from 1^st^ November 2009 to 6^th^ December 2012 that have been registered in the International Plant Name Index (as per 6^th^ December 2011, data provided by Christine Barker, IPNI).

	**All ranks**	**Genus rank**	**Infrageneric rank**	**Specific rank**	**Infraspecific rank**
2010	11	1	0	10	0
2011	41	2	0	38	1
2012	44	1	1	42	0
**Total**	**96**	**4**	**1**	**90**	**1**

**Table 3.** Other nomenclatural novelties published in PhytoKeys for the entire period of its existence (data provided by Christine Barker, IPNI).

	**New combinations**			**New names**
	**All ranks**	**Specific rank**	**Infraspecific rank**	**Specific rank**
2010	1	1	0	0
2011	43	43	1	8
2012	11	5	6	2
**Total**	**55**	**49**	**6**	**10**

PhytoKeys has always been engaged with open data publishing. One of the pioneering methods for data publishing converted a conventional floristic checklist, written in a standard word processing program, into structured data in the Darwin Core Archive format (Remsen et al. 2012). A manuscript (De Egea et al. 2012) consisting of more than 4,100 taxon names, was submitted to PhytoKeys as a Microsoft Word file. After peer-review and editorial acceptance, the final revised version was converted into the Darwin Core Archive format from the original manuscript and published both as a conventional paper in PhytoKeys and as DwC-A structured data through the Global Biodiversity Information Facility (GBIF) Integrated Publishing Toolkit (IPT). In addition, and for the convenience of the readers and data users, the same data were also published as a supplementary Excel file in an Appendix to the checklist (doi: 10.3897/phytokeys.9.2279.app1). After publication, the data became available through the GBIF infrastructure in order to be re-used on their own or collated with other data.

The first data paper in PhytoKeys was published in May 2012 by Van Landuyt et al. (2012). It described “Florabank1” – a database comprising distributional data on the wild plants of Flanders and the Brussels Capital Region of Belgium.

The top ten papers in PhytoKeys through the 5^th^ of December 2012 have been accessed 55,502 times (Table 4). The article by Popovkin et al. (2011) on a new, genuflexing plant from Brazil took the lead as the most downloaded paper by reaching more than 10,200 views in 14 months. Among the top ten most viewed articles, the paper by Miller et al. (2011), a review article on the outcomes of the Botanical Nomenclature section at the XVIII International Botanical Congress and its Spanish version (Knapp et al. 2011b), was downloaded more than 12,500 times

**Table 4.** The top ten most viewed articles of PhytoKeys according to the PhytoKeys website counter accessed on the 5^th^ of December 2012.

**Article**	**Page views**
Popovkin et al. 2011 – *Spigelia genuflexa* (Loganiaceae), a new geocarpic species from northeastern Bahia, Brazil	10,212
Miller et al. 2011 – Outcomes of the 2011 Botanical Nomenclature Section at the XVIII International Botanical Congress	8,657
Knapp et al. 2011a – Changes to publication requirements made at the XVIII International Botanical Congress in Melbourne - what does e-publication mean for you?	5,560
Telford et al. 2011 – A new Australian species of *Luffa* (Cucurbitaceae) and typification of two Australian *Cucumis* names, all based on specimens collected by Ferdinand Mueller in 1856	5,277
Kress et al. 2010 – *Larsenianthus*, a new Asian genus of Gingers (Zingiberaceae) with four species	5,155
Penev et al. 2010a – Fast, linked, and open – the future of taxonomic publishing for plants: launching the journal PhytoKeys	4,872
Vallejo-Marín 2012 - *Mimulus peregrinus* (Phrymaceae): A new British allopolyploid species	4,411
Knapp et al. 2011b - Translation into Spanish of: “Changes to publication requirements made at the XVIII International Botanical Congress in Melbourne - what does e-publication mean for you?”. Translated by Carmen Ulloa Ulloa, Lourdes Rico Arce, and Renée H. Fortunato	3,878
Knapp 2010 - New species of *Solanum* (Solanaceae) from Peru and Ecuador	3,801
Tepe et al. 2012 – A new species of *Solanum* named for Jeanne Baret, an overlooked contributor to the history of botany	3,679

In order to promote plant taxonomy, Pensoft’s Public Relations office has initiated a new service aimed at facilitating our authors in promoting their research among the general public and science media. Since its launch, altogether 17 international press releases have been prepared and distributed through EurekAlert!, one of the world largest online distributors of science news, supplying information to more than 7,500 mass media and independent science journalists, and Pensoft’s own channels (see Table 5 for the top ten most accessed press releases of PhytoKeys articles). At the top, with approxmately 5,000 views, is the press release of the article by Vallejo-Marín (2012) describing a new monkey flower, *Mimulus peregrinus* (Phrymaceae) discovered on the bank of a stream in Scotland created by the union of two foreign plant species. This paper described a rare example of a new species that originated in the wild in the last 150 years.

**Table 5.** The top ten most accessed press releases of PhytoKeys articles posted through EurekAlert!. The counter registers only downloads from EurekAlert!, mostly by science media and journalists. The actual number of readers is most likely much higher than this number.

	Title	Author/s and year of publication of the original article	Date posted	Page views since posted
1.	Rare glimpse into the origin of species: Plant overcomes infertility to give rise to a new species in Scotland	Vallejo-Marín 2012	10-Jul-2012	4,847
2.	Brave new world: Pioneering electronic publication of new plant species	Vorontsova and Knapp 2012	1-Jan-2012	3,424
3.	A new wild ginger discovered from the evergreen forest of Western Ghats of South India	Thomas et al. 2012	6-Jan-2012	3,181
4.	Jeanne Baret, botanist and first female circumnavigator, finally commemorated in name of new species	Tepe et al. 2012	3-Jan-2012	3,092
5.	Early lineage of Larkspur and Monkshood plants rediscovered in Southern Europe	Jabbour and Renner 2011	8-Dec-2011	2,767
6.	Plant DNA speaks English, identifies new species	Filipowicz et al. 2012	23-Mar-2012	2,634
7.	Early land plants: Early adopters!: The first electronically described liverwort species comes from New Zealand	von Konrat et al. 2012	4-Jan-2012	2,474
8.	Marquesas Islands in French Polynesia yield 18 new species of rare ferns and flowering plants	Tronchet and Lowry II 2011	19-Jul-2011	2,119
9.	Revolutionary changes to the Botanical Code published in 16 journals and 5 languages	Knapp et al. 2011a	14-Sep-2011	1,252
10.	Botany student proves ‘New England Banksia’ a distinct species	Stimpson et al. 2012	28-Aug-2012	1,195

In conclusion, PhytoKeys, along with its 'brother' journals Zookeys and MycoKeys, continues to evolve its editorial workflow, constantly implementing new and improved publishing and dissemination technologies, thus always being on point for digital biodiversity science. We would like to thank all of our authors, reviewers, subject editors, readers, and journalistic followers without whose support PhytoKeys would not have become such a successful journal in just two years time! We also thank Christine Barker (IPNI) for providing information on the nomenclatural novelties published in PhytoKeys that have been registered in IPNI.
